# Identification of Phenolic Compounds-Rich Grape Pomace Extracts Urine Metabolites and Correlation with Gut Microbiota Modulation

**DOI:** 10.3390/antiox7060075

**Published:** 2018-06-04

**Authors:** Stéphanie Chacar, Mehrad Tarighi, Nassim Fares, Jean-François Faivre, Nicolas Louka, Richard G. Maroun

**Affiliations:** 1 Centre d’Analyses et de Recherche, UR GPF, Laboratoire CTA, Faculté des Sciences, Université Saint-Joseph, B.P. 11-514 Riad El Solh, Beirut 1107 2050, Lebanon; stephanie.chacar@net.usj.edu.lb (S.C.); nicolas.louka@usj.edu.lb (N.L.); 2 Laboratoire de Recherche en Physiologie et Physiopathologie, LRPP, Pôle Technologie Santé, Faculté de Médecine, Université Saint Joseph, B.P. 11-514 Riad El Solh, Beirut 1107 2050, Lebanon; nassim.fares@usj.edu.lb; 3 Laboratoire Signalisation et Transports Ioniques Membranaires (STIM), Université de Poitiers EA 7349, 86000 Poitiers, France; jean-francois.faivre@univ-poitiers.fr; 4 Institut de Chimie des Milieux et Matériaux de Poitiers (IC2MP), Université de Poitiers UMR CNRS 7285, 86000 Poitiers, France; mehrad.tarighi@univ-poitiers.fr

**Keywords:** gut microbiota, antioxidants, phenolic compounds, urinary metabolites, aging

## Abstract

The high diversity of phenolic compounds (PC) found in food matrices makes it challenging to analyze their bioavailability and their impact on health and functional metabolism. It is well recognized that PC do modulate the composition of the gut microbiota (GM), however, the literature still lacks significant data concerning the link between the metabolic fate of the ingested compounds and their bioactivity, mainly when considering the secondary metabolites produced. In this study, we assessed the metabolic fate of PC for a period covering 14 months of daily intake to identify the metabolites that could be responsible for the effects of PC on the GM observed in our previous work. Urinary analysis of polyphenol metabolites was performed using a high resolution mass spectrometry LC-QTOF-MS method. Among the sixteen metabolites identified, 3-hydroxyphenylacetic acid and 2-(4-hydroxyphenyl) propionic acid were detected simultaneously and, therefore, correlated with the growth of *Bifidobacterium* in the rat GM. In addition, Daidzedin, detected only at 14 months post-treatment, mostly interfered with the growth inhibition of *Clostridium* (Cluster I). In conclusion, the impact of the long-term intake of PC on rat GM seems to be related to specific metabolites produced after ingestion of the parental compounds and this may also be due to their additional synergistic effects.

## 1. Introduction

Presently, it is common to hear about the importance of the daily consumption of phenolic compounds (PC) from food matrices and the obvious medicinal and health benefits they exert. Several studies have investigated the metabolism of these compounds in order to know the proportion of the compound that will act in the body as opposed to the absorbed amount [1]. Following the ingestion of PC, 5–10% are absorbed by the small intestine and the remaining amount reaches the colon where it will undergo a hydrolysis process by the gut microbiota (GM) [2]. Next, the produced aglycones are metabolized and undergo conjugation processes, such as sulfation, methylation, and glucuronidation [3]. It is now recognized that the biotransformation of PC into their metabolites by GM increases their bioavailability, however, they barely reach micromolar concentrations in plasma and urine [4]. In addition, other studies concentrated on the modulation of the composition of the gut microbial community by PC, which takes part in the two-mutual reaction between PC and GM. In a previous work, we analyzed the rat gut microbiota composition following a long-term intake of PC rich grape pomace extracts. We found that *Bifidobacterium* growth was significantly higher than in the control group. *Lactobacillus* decreased with time in all the treated and untreated groups. *Bacteroides*, *Clostridium leptum* subgroup (*Clostridium* Cluster IV), and *Enterococcus* were not significantly changed by PC intake when compared to the control group. Nevertheless, after 14 months of treatment PC abolished the increase of *Clostridium* (Cluster I) observed in the control group. Thus, we clearly demonstrated that PC do selectively modulate rat gut microbiome to a healthier phenotype in long-term feeding rats. In fact, our results showed that long-term PC intake inhibited the growth of non-beneficial bacteria, such as *Clostridium* (Cluster I), and enhanced the growth of probiotic ones, such as the *Bifidobacterium* strain [5]. In this study, we were interested in identifying the metabolites produced following the consumption of the mixture of grape pomace for 14 months, regardless of their quantification. Additionally, we also aimed to establish a link between the metabolic fate of these compounds and their bioactivity on the GM studied in our previous work.

## 2. Short Methods

PC extraction was performed as previously described [5]. Briefly, the PC were extracted from grape pomace extracts via a solid liquid extraction, then the solutions were spray dried and a powder was obtained consisting of a pure mixture of PC. 

Thirty male adult rats were used and the protocols were designed according to the Guiding Principles in the Care and Use of Animals approved by the Council of the American Physiological Society, and were in adherence to the *Guide for the Care and Use of Laboratory Animals* published by the U.S. Natl. Inst. of Health (NIH Publication no. 85–23, revised 1996) and according to the European Parliament Directive 2010/63 EU. The study was approved by the ethical committee of Saint Joseph University of Beirut. The ethical approval number of our project is: USJ-215-46. The rats were divided into 5 groups randomly (*n* = 6 per group), which were given daily over 14 months, in the drinking water, different concentrations of PC: 2.5, 5, 10, and 20 mg/kg/day (for PC 2.5, PC 5, PC 10, and PC 20, respectively) or 0.1% dimethyl sulfoxide (DMSO vehicle (control group)). Quantification of total PC prior to their administration was achieved as described by [5]. 

### Urine Analysis by LC-QTOF Mass Spectrometry

Urine samples were collected at baseline from young untreated rats (2 months old) and from rats fed daily with PC or vehicle at 6 and 14 months post-treatment. The collected urine samples were diluted with milli-Q water 1:10 and then filtered through a 0.22 μm filter. 

The characterization of the samples was performed with an Ultra-performance liquid chromatography with a QTOF mass spectrometer (UPLC/MS) (Bruker Maxis Impact HD) equipped with an electrospray source operating in negative ion and set to scan the metabolites in a range of *m*/*z* from 50 to 1000. The urinary metabolites separation was realized on an Acquity UPLC C18 column, 1.7 μm, 2.1 × 100 mm (Waters, Milford, MA, USA), with an Acquity UPLC C18 VanGuard pre-column, 2.1 × 5 mm, 1.7 μm (Waters, Milford, MA, USA). The source temperature was set at 200 °C, with a cone gas flow of 500 μL·h^−1^; a desolvation gas temperature of 300 °C; and a nebulization gas flow of 400 L·h^−1^. The injection volume was 10 µL. Mobile phase A, consisting of 0.1% formic acid in Ultra-pure water, and mobile phase B, consisting of 0.1% formic acid in methanol, were used. The elution profile had the following proportions (*v*/*v*) of solvent: 95–5% of solvent A to 5–95% of solvent B during 10 min. The QTOF was calibrated with Na formate and acetate. Accurate mass measurements (error < 5 ppm for analytes) were obtained by means of an automated calibrant delivery system using a dual-nebulizer electrospray ionization (ESI) source. 

## 3. Results

### Identification of Urinary Metabolites Following Grape Pomace Extracts Consumption

**Figure 1:**
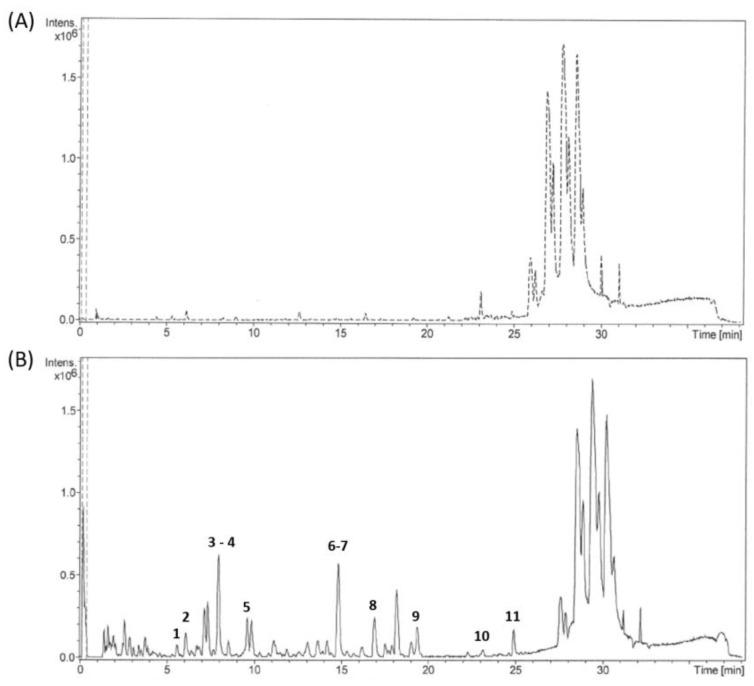
Liquid chromatography/Mass spectrometry LC/MS chromatogram for rats’ urine samples: (**A**) Control untreated rat and (**B**) PC 5 mg/kg/day treated rat after 14 months of treatment. The identified metabolites in chromatogram B are: 1. Tyrosol, 2. Pyrocatechol-sulfate, 3. Homovanillic acid-sulfate, 4. Ferulic acid, 5. Daidzedin-sulfate, 6. Benzoic acid-sulfate, 7. 3-hydroxyphenylacetic acid-sulfate, 8. Daidzedin, 9. 3-hydroxyphenylacetic acid-methyl, 10. trans-cinnamic acid, and 11. 2-(4-hydroxyphenyl) propionic acid-glucuronide.

In this work, sixteen PC metabolites were identified in the urine of the treated rats with PC-rich grape pomace. Their detection was based on a large database comprising of all forms of PC metabolites found in the literature as native form or conjugated. For an optimal characterization, the metabolites found in the urine samples of young and untreated control rats were subtracted and, thus, eliminated from the analysis. Amongst the 16 detected metabolites, a total of 7 [M − H]^−^ metabolites, 7 sulfated, 1 methylated, and 1 glucuronidated, were identified. The detected PC metabolites in the urine of all the treated rats, after 6 and 14 months of treatment, are summarized in Table 1. Figure 1 shows two representative chromatograms, (A) for control untreated rat and (B) for PC 5 mg/kg/day treated rat, after 14 months of treatment in which 11 PC metabolites were identified. 

In our previous work, we studied the long-term impact of PC-rich grape pomace on rat GM and we found that the mixture of PC can inhibit non beneficial bacteria from the rat microbiota and potentiate the growth of probiotic ones [5]. During this work, we aimed to reveal the identity of the compounds and metabolites that could be responsible for the selective modulation of the rat gut microbiome. In fact, the biotransformation of PC depends on both their structure and the composition of the GM. It has been shown that particular species with certain genes for specific enzymes interfere in the biotransformation of PC, and the modulation of the GM depends on the resultant metabolites [6]. 

In our study, some metabolites were detected in the urine during the entirety of the treatment period (e.g., homivanillic acid, ferulic acid, and benzoic acid) and others fluctuated within the treatment (e.g., coumaric acid, genistein, and valerolactone). 3-hydroxyphenylacetic acid and 2-(4-hydroxyphenyl) propionic acid were detected simultaneously and, therefore, correlated with the growth of *Bifidobacterium* in the rat GM as already observed [5]. In addition, the presence of Daidzedin metabolite, the phenolic sulfate derivative detected in the urine of all treated rats at 14 months post-treatment, could be responsible for the growth inhibition of *Clostridium* (Cluster I) that was observed after the same period of treatment. 

## 4. Discussion

From literature data results, Tzounis et al., showed an increase in the growth of *Bifidobacterium* after inoculation with flavonols [7]. Furthermore, a 50 mg/kg dose of de-alcoholized wine polyphenols consumed by carcinogen-treated rats for 16 weeks was associated with an increase of Bifidobacteria in the colon content [8]. In addition, Gwiazdowska et al., reported that some derivatives of hydroxycinnamic and hydroxybenzoic acids enhanced the growth of *Bifidobacterium* in vitro [9]. In our work, we tried to correlate the presence of certain metabolites with the inhibition or the activation of GM growth. This could lead to a better understanding of PC metabolites’ mechanism of action, in vivo. The detection of 3-hydroxyphenylacetic acid and 2-(4-hydroxyphenyl) propionic acid, simultaneously, with the growth of *Bifidobacterium* after consumption of PC 2.5 and PC 5 support the in vitro results of Gwiazdowska, highlighting that phenolic acid derivatives enhance the growth of *Bifidobacterium*. These two metabolites could be mostly responsible for the growth of *Bifidobacterium* as they were not detected in the urine samples of rats fed with higher concentrations of PC (PC 10 and PC 20), where no increase was observed of the *Bifidobacterium*. The nonadditional effect observed at high concentrations could be related to a mechanism of saturation of PC derivatives in plasma [10]. 

As for *Clostridium* (Cluster I), the metabolic end products of caffeic acid (CFA) and chlorogenic acid (CGA) did not inhibit the growth of the pathogenic bacteria, *Clostridium perfringens*, although the main precursors, CFA and CGA, were able to inhibit its growth during the minimum inhibitory concentration (MIC) testing at 300 and 400 ppm, respectively [11]. Antimicrobial activity of curcumin, coumarin, ellagic acid, (-) epicatechin, and others have been reported following an MIC test for these compounds during 60 h of incubation [12]. In our work, the absence of Daidzedin sulfate derivatives coincides with the elevated levels of *Clostridium* in all groups, and its detection in the urine of months rats treated for 14 months accords with the growth inhibition of *Clostridium* (Cluster I). To our knowledge, we are the first to identify an in vivo metabolite whose presence positively modulates GM. This effect could also be due to its synergistic action with other metabolic end products identified in this work. Although no direct correlation was established between the presence of thirteen out of the sixteen metabolites identified in this work and the rat GM modulation, these compounds may have synergistically exerted their effects as it was demonstrated in other studies. Dong et al., reported that a combination of daidzein and genistein was more effective in inducing apoptosis and inhibiting proliferation in prostate cancer cells than individual soy isoflavones at equivalent concentrations [13]. Additionally, malvidin-3-glucoside mixed with other anthocyanins exhibited a synergistic effect in promoting beneficial microbes [14]. However, the exact mechanism of the synergistic effect that occurred remains poorly defined. 

## 5. Conclusions

The impact of long-term intake of PC on rat GM seems to be attributed, in part, to specific produced metabolites. The presence of both 3-hydroxyphenylacetic acid and 2-(4-hydroxyphenyl) propionic acid was directly correlated with the growth of *Bifidobacterium*. As for the phenolic sulfate derivative of Daidzedin, it was detected in the urine of rats at 14 months post-treatment. This perfectly correlates with the period when *Clostridium perfringens* growth was inhibited. It is to be mentioned that the occurred effects on the GM may also be due to their additional synergistic collaboration with other biotransformed compounds. The quantification of the identified metabolites and the characterization of PC-rich grape pomace metabolites in plasma would be valuable in better understanding their biological health effects. 

## Figures and Tables

**Table 1:** Excretion of phenolic compounds (PC) metabolites in the urine after 6 and 14 months of consumption of PC-rich grape pomace mixture.

Compound Structure	PC Metabolites	PC 2.5		PC 5		PC 10		PC 20	
		6 mo pt	14 mo pt	6 mo pt	14 mo pt	6 mo pt	14 mo pt	6 mo pt	14 mo pt
M-1 derivatives	Coumaric acid	−	−	+	−	+	−	−	−
	Genistein	+	−	+	−	+	+	+	+
	Daidzedin	+	+	+	+	+	+	+	+
	Valerolactone	+	−	−	−	−	+	−	−
	Tyrosol	−	−	−	+	−	−	−	−
	Trans-cinnamic acid	−	−	−	+	−	−	−	+
	Apigenin	−	−	−	−	−	−	−	+
Phenolic sulfate derivatives	Pyrocatechol	+	+	+	+	+	+	+	+
	3-hydroxyphenylacetic acid	+	+	+	+	+	+	+	+
	Homovanillic acid	+	+	+	+	+	+	+	+
	Ferulic acid	+	+	+	+	+	+	+	+
	Enterolactone	−	−	−	−	−	−	−	+
	Benzoic acid	+	+	+	+	+	+	+	+
	Daidzedin	−	+	−	+	−	+	−	+
Phenolic methyl derivatives	3-hydroxyphenylacetic acid	+	+	+	+	−	−	−	−
Phenolic glucuronide derivatives	2-(4-hydroxyphenyl) propionic acid	+	+	+	+	−	−	−	−

−: absence of PC metabolite; +: presence of PC metabolite.
